# 4-Hydroxy­ethyl-4-methyl­morpholinium chloride

**DOI:** 10.1107/S1600536808030195

**Published:** 2008-09-27

**Authors:** Wang Mei-Ling, Zang Hong-Jun, Cheng Bo-Wen, Jun Song

**Affiliations:** a School of Materials and Chemical Engineering and Key Laboratory of Hollow Fiber Membrane Materials & Membrane Processes, Tianjin Polytechnic University, Tianjin 300160, People’s Republic of China

## Abstract

In the title compound, C_7_H_16_NO_2_^+^·Cl^−^, the asymmetric unit consists of two cation–anion pairs, in which the ion pairs are inter­connected by weak C—H⋯Cl hydrogen bonds. Each cation forms a network of weak C—H⋯Cl hydrogen bonds to surrounding chloride ions. The morpholine ring is in a chair conformation. The crystal structure is consolidated by O—H⋯Cl, C—H⋯Cl and C—H⋯O inter­molecular hydrogen bonding.

## Related literature

For general background, see: Abedin *et al.* (2004 ▶, 2005 ▶); Kim *et al.* (2005 ▶, 2006 ▶).
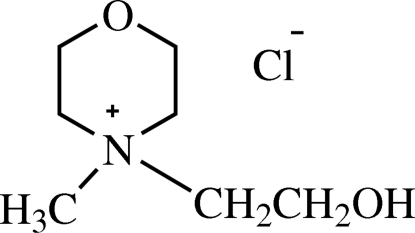


## Experimental

### 

#### Crystal data

C_7_H_16_NO_2_^+^·Cl^−^
*M* *_r_* = 181.66
Orthorhombic, 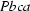

*a* = 12.181 (2) Å
*b* = 12.452 (3) Å
*c* = 23.856 (5) Å
*V* = 3618.5 (13) Å^3^
*Z* = 16
Mo *K*α radiation
μ = 0.38 mm^−1^
*T* = 113 (2) K
0.16 × 0.12 × 0.10 mm

#### Data collection

Rigaku Saturn diffractometer
Absorption correction: multi-scan (*CrystalClear*; Rigaku/MSC 2005 ▶) *T*_min_ = 0.942, *T*_max_ = 0.963
19758 measured reflections
3198 independent reflections
2938 reflections with *I* > 2σ(*I*)
*R*_int_ = 0.036

#### Refinement

*R*[*F* ^2^ > 2σ(*F* ^2^)] = 0.030
*wR*(*F* ^2^) = 0.076
*S* = 1.06
3198 reflections
203 parameters
H-atom parameters constrained
Δρ_max_ = 0.23 e Å^−3^
Δρ_min_ = −0.22 e Å^−3^

### 

Data collection: *CrystalClear* (Rigaku/MSC, 2005 ▶); cell refinement: *CrystalClear*; data reduction: *CrystalClear*; program(s) used to solve structure: *SHELXS97* (Sheldrick, 2008 ▶); program(s) used to refine structure: *SHELXL97* (Sheldrick, 2008 ▶); molecular graphics: *SHELXTL* (Sheldrick, 2008 ▶); software used to prepare material for publication: *SHELXTL*.

## Supplementary Material

Crystal structure: contains datablocks I, global. DOI: 10.1107/S1600536808030195/si2107sup1.cif

Structure factors: contains datablocks I. DOI: 10.1107/S1600536808030195/si2107Isup2.hkl

Additional supplementary materials:  crystallographic information; 3D view; checkCIF report

## Figures and Tables

**Table 1:** Hydrogen-bond geometry (Å, °)

*D*—H⋯*A*	*D*—H	H⋯*A*	*D*⋯*A*	*D*—H⋯*A*
O2—H2⋯Cl2^i^	0.82	2.20	3.0222 (13)	178
O4—H4⋯Cl1	0.82	2.24	3.0505 (13)	168
C1—H1*B*⋯Cl1^ii^	0.97	2.64	3.5812 (16)	165
C2—H2*B*⋯O2^iii^	0.97	2.59	3.335 (2)	134
C4—H4*B*⋯Cl2^iv^	0.97	2.72	3.6390 (17)	158
C5—H5*C*⋯Cl1^v^	0.96	2.71	3.6527 (16)	167
C6—H6*A*⋯Cl1^iv^	0.97	2.77	3.6888 (18)	158
C8—H8*A*⋯O2	0.97	2.45	3.385 (2)	163
C9—H9*B*⋯Cl1^iv^	0.97	2.80	3.7625 (17)	174
C11—H11*A*⋯O4	0.97	2.52	3.064 (2)	115
C13—H13*A*⋯Cl2^vi^	0.97	2.80	3.7009 (18)	154
C13—H13*B*⋯O3^vii^	0.97	2.60	3.3013 (19)	130

Symmetry codes: (i) 
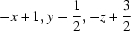
; (ii) 
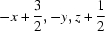
; (iii) 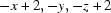; (iv) 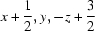; (v) 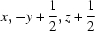; (vi) 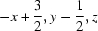; (vii) 
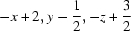
.

**Fig. 1.**
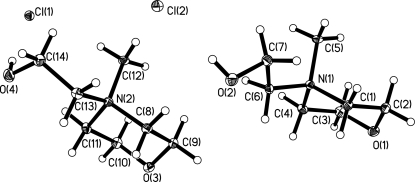
A view of the molecular structure of (I), showing the atom-numbering scheme. Dispacement ellipsoids are drawn at the 30% probability level.

**Fig. 2.**
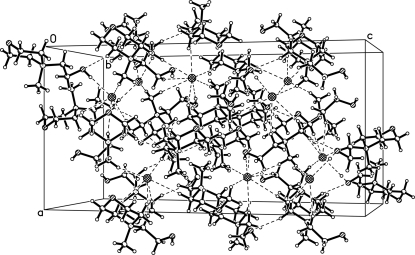
A section of the the crystal packing viewed approximately down [010], showing hydrogen-bond interactions as dashed lines. H atoms are shown as small spheres of arbitary radii.

## supplementary crystallographic information

### Comment

Quaternary morpholine halides are valuable precursors for the preparation of ionic liquids (ILs) by ion metathesis (Kim *et al.*,2005). The excellent conductivity, broad electrochemical window, thermal stability, and low volatility of ILs have made them promising media for electrochemical processes (Abedin *et al.*,2004; Abedin *et al.*,2005). In particular, ILs based on the morpholinium cation are favored becaused of their low cost, easy synthesis, and electrochemical stability (Kim *et al.*,2006). So far, only a few crystallographic studies have been performed on salts. We report here a new example structure of this class.

The molecular structure of (I) is illustrated in Fig. 1. For the title compound two crystallographically independent molecules are present in the asymmetric unit of the cell. The morpholine unit adopts a chair conformation. The bond distances and angles in the cation are normal within experimental error.

The crystal packing of (I) is illustrated in Fig. 2. The Cl^-^anion involved in forming weak C—H···Cl hydrogen bonds. Each cation forms a network of weak C—H···Cl hydrogen bonds to surrounding chloride ions. The cation/anion pairs are interconnected by weak H—Cl bonding. The O atom of the hydroxyl group in the molecule involved in forming O—H···Cl and weak C—H···O hydrogen bonds. The crystal structure is consolidated by O—H···Cl, C—H···Cl and C—H···O intermolecular hydrogen bonding.

### Experimental

Under vigorous stirring, 2-chloroethanol(0.12 mol) was added to a solution of 4-methylmorpholine(0.1 mol) in 20 mL of acetonitrile. The mixture was stirred at 85 °C for 35 h. The solvent was removed under reduced pressure. The remaining brownish, viscous liquid crystallized slowly at room temperature in ethanol and acetone [1/20(*v*/*v*)]. A single-crystal was obtained by slow evaporation of a solution in ethanol and acetone [1/20(*v*/*v*)].

### Refinement

The H atoms bonded to C and O atoms were included in the refinement in the riding and rotation model approximation, with C–H = 0.96–0.97 Å, O–H = 0.82 Å, and *U*_iso_ (H) = 1.2 *U*_eq_ (C, O atom). For the H atoms attached to C atoms of methyl groups, their *U*_iso_(H) = 1.5*U*_eq_(C).

### Crystal data

**Table:**

C_7_H_16_NO_2_^+^·Cl^−^	*D*_x_ = 1.334 Mg m^−^^3^
*M**_r_* = 181.66	Mo *K*α radiation, λ = 0.71073 Å
Orthorhombic, *P**b**c**a*	Cell parameters from 8950 reflections
*a* = 12.181 (2) Å	θ = 1.9–27.9°
*b* = 12.452 (3) Å	µ = 0.38 mm^−^^1^
*c* = 23.856 (5) Å	*T* = 113 K
*V* = 3618.5 (13) Å^3^	Prism, colorless
*Z* = 16	0.16 × 0.12 × 0.10 mm
*F*(000) = 1568	

### Data collection

**Table:**

Rigaku Saturn diffractometer	3198 independent reflections
Radiation source: rotating anode	2938 reflections with *I* > 2σ(*I*)
confocal	*R*_int_ = 0.036
ω scans	θ_max_ = 25.0°, θ_min_ = 2.4°
Absorption correction: multi-scan (CrystalClear; Rigaku/MSC 2005)	*h* = −11→14
*T*_min_ = 0.942, *T*_max_ = 0.963	*k* = −14→14
19758 measured reflections	*l* = −23→28

### Refinement

**Table:**

Refinement on *F*^2^	Primary atom site location: structure-invariant direct methods
Least-squares matrix: full	Secondary atom site location: difference Fourier map
*R*[*F*^2^ > 2σ(*F*^2^)] = 0.030	Hydrogen site location: inferred from neighbouring sites
*wR*(*F*^2^) = 0.076	H-atom parameters constrained
*S* = 1.06	*w* = 1/[σ^2^(*F*_o_^2^) + (0.0348*P*)^2^ + 1.9139*P*] where *P* = (*F*_o_^2^ + 2*F*_c_^2^)/3
3198 reflections	(Δ/σ)_max_ = 0.002
203 parameters	Δρ_max_ = 0.23 e Å^−^^3^
0 restraints	Δρ_min_ = −0.22 e Å^−^^3^

### Special details

**Table:**

Geometry. All e.s.d.'s (except the e.s.d. in the dihedral angle between two l.s. planes) are estimated using the full covariance matrix. The cell e.s.d.'s are taken into account individually in the estimation of e.s.d.'s in distances, angles and torsion angles; correlations between e.s.d.'s in cell parameters are only used when they are defined by crystal symmetry. An approximate (isotropic) treatment of cell e.s.d.'s is used for estimating e.s.d.'s involving l.s. planes.
Refinement. Refinement of *F*^2^ against ALL reflections. The weighted *R*-factor *wR* and goodness of fit *S* are based on *F*^2^, conventional *R*-factors *R* are based on *F*, with *F* set to zero for negative *F*^2^. The threshold expression of *F*^2^ > σ(*F*^2^) is used only for calculating *R*-factors(gt) *etc*. and is not relevant to the choice of reflections for refinement. *R*-factors based on *F*^2^ are statistically about twice as large as those based on *F*, and *R*- factors based on ALL data will be even larger.

### Fractional atomic coordinates and isotropic or equivalent isotropic displacement parameters (Å^2^)

**Table:**

	*x*	*y*	*z*	*U*_iso_*/*U*_eq_	
Cl1	0.68479 (3)	0.09749 (3)	0.579742 (15)	0.01752 (11)	
Cl2	0.30866 (3)	0.32732 (3)	0.691066 (15)	0.01992 (11)	
O1	0.99622 (9)	0.31527 (9)	1.03992 (5)	0.0231 (3)	
O2	0.86105 (10)	−0.08159 (9)	0.88818 (5)	0.0231 (3)	
H2	0.8137	−0.1060	0.8673	0.035*	
O3	1.09148 (9)	0.15192 (9)	0.74353 (4)	0.0211 (3)	
O4	0.84900 (10)	−0.08501 (10)	0.59544 (5)	0.0265 (3)	
H4	0.8108	−0.0332	0.5870	0.040*	
N1	0.87164 (10)	0.18106 (9)	0.96226 (5)	0.0139 (3)	
N2	0.91549 (10)	0.00400 (9)	0.71337 (5)	0.0144 (3)	
C1	0.92450 (13)	0.13932 (12)	1.01538 (6)	0.0164 (3)	
H1A	0.9974	0.1125	1.0068	0.020*	
H1B	0.8814	0.0801	1.0300	0.020*	
C2	0.93275 (14)	0.22648 (12)	1.05946 (6)	0.0201 (3)	
H2A	0.8596	0.2510	1.0692	0.024*	
H2B	0.9664	0.1972	1.0930	0.024*	
C3	0.94589 (14)	0.36040 (13)	0.99131 (7)	0.0211 (3)	
H3A	0.9889	0.4212	0.9785	0.025*	
H3B	0.8732	0.3863	1.0009	0.025*	
C4	0.93669 (13)	0.27905 (12)	0.94444 (6)	0.0177 (3)	
H4A	1.0097	0.2570	0.9330	0.021*	
H4B	0.9012	0.3122	0.9124	0.021*	
C5	0.75259 (13)	0.20764 (13)	0.97066 (6)	0.0183 (3)	
H5A	0.7147	0.1455	0.9846	0.027*	
H5B	0.7209	0.2292	0.9356	0.027*	
H5C	0.7460	0.2652	0.9972	0.027*	
C6	0.88210 (13)	0.09905 (11)	0.91578 (6)	0.0160 (3)	
H6A	0.9587	0.0786	0.9125	0.019*	
H6B	0.8607	0.1327	0.8808	0.019*	
C7	0.81376 (13)	−0.00259 (12)	0.92364 (7)	0.0191 (3)	
H7A	0.8160	−0.0259	0.9624	0.023*	
H7B	0.7379	0.0104	0.9133	0.023*	
C8	0.98190 (13)	−0.00380 (12)	0.76704 (6)	0.0178 (3)	
H8A	0.9364	−0.0341	0.7964	0.021*	
H8B	1.0436	−0.0518	0.7611	0.021*	
C9	1.02402 (13)	0.10464 (12)	0.78564 (6)	0.0197 (3)	
H9A	0.9624	0.1516	0.7935	0.024*	
H9B	1.0661	0.0965	0.8199	0.024*	
C10	1.02888 (14)	0.16834 (12)	0.69399 (6)	0.0204 (3)	
H10A	1.0744	0.2029	0.6659	0.024*	
H10B	0.9677	0.2157	0.7022	0.024*	
C11	0.98544 (13)	0.06294 (12)	0.67078 (6)	0.0180 (3)	
H11A	0.9420	0.0772	0.6375	0.022*	
H11B	1.0467	0.0177	0.6600	0.022*	
C12	0.80896 (13)	0.06048 (13)	0.72421 (7)	0.0188 (3)	
H12A	0.8231	0.1282	0.7421	0.028*	
H12B	0.7639	0.0170	0.7482	0.028*	
H12C	0.7716	0.0724	0.6893	0.028*	
C13	0.89419 (14)	−0.11115 (12)	0.69480 (6)	0.0186 (3)	
H13A	0.9637	−0.1424	0.6835	0.022*	
H13B	0.8683	−0.1513	0.7270	0.022*	
C14	0.81313 (14)	−0.12733 (13)	0.64760 (7)	0.0228 (4)	
H14A	0.7994	−0.2036	0.6432	0.027*	
H14B	0.7442	−0.0934	0.6577	0.027*	

### Atomic displacement parameters (Å^2^)

**Table:**

	*U*^11^	*U*^22^	*U*^33^	*U*^12^	*U*^13^	*U*^23^
Cl1	0.0183 (2)	0.0171 (2)	0.01717 (19)	−0.00094 (14)	0.00115 (14)	−0.00020 (13)
Cl2	0.0178 (2)	0.0216 (2)	0.0204 (2)	0.00114 (15)	0.00057 (14)	0.00214 (14)
O1	0.0190 (6)	0.0245 (6)	0.0257 (6)	−0.0032 (5)	−0.0024 (5)	−0.0059 (5)
O2	0.0225 (7)	0.0207 (6)	0.0262 (6)	−0.0008 (5)	−0.0019 (5)	−0.0075 (5)
O3	0.0177 (6)	0.0230 (6)	0.0225 (6)	−0.0025 (5)	−0.0016 (4)	−0.0008 (4)
O4	0.0276 (7)	0.0292 (7)	0.0226 (6)	0.0092 (5)	−0.0014 (5)	−0.0059 (5)
N1	0.0131 (7)	0.0147 (6)	0.0138 (6)	−0.0007 (5)	0.0002 (5)	0.0009 (5)
N2	0.0141 (7)	0.0139 (6)	0.0151 (6)	0.0009 (5)	0.0005 (5)	−0.0001 (5)
C1	0.0165 (8)	0.0172 (8)	0.0155 (7)	0.0018 (6)	−0.0024 (6)	0.0021 (6)
C2	0.0197 (9)	0.0225 (8)	0.0183 (8)	0.0014 (7)	−0.0019 (6)	−0.0015 (6)
C3	0.0218 (9)	0.0164 (8)	0.0252 (8)	−0.0022 (7)	0.0028 (7)	−0.0009 (6)
C4	0.0184 (9)	0.0160 (8)	0.0188 (8)	−0.0038 (6)	0.0037 (6)	0.0026 (6)
C5	0.0105 (8)	0.0225 (8)	0.0220 (8)	0.0026 (6)	−0.0005 (6)	−0.0007 (6)
C6	0.0176 (8)	0.0173 (8)	0.0133 (7)	−0.0005 (6)	0.0013 (6)	−0.0026 (6)
C7	0.0188 (9)	0.0196 (8)	0.0188 (8)	−0.0023 (6)	0.0012 (6)	−0.0029 (6)
C8	0.0163 (8)	0.0217 (8)	0.0153 (8)	0.0035 (6)	−0.0011 (6)	0.0042 (6)
C9	0.0177 (9)	0.0259 (8)	0.0154 (7)	0.0024 (7)	−0.0013 (6)	−0.0021 (6)
C10	0.0227 (9)	0.0182 (8)	0.0202 (8)	−0.0023 (7)	0.0006 (6)	0.0026 (6)
C11	0.0217 (9)	0.0189 (8)	0.0135 (7)	−0.0023 (7)	0.0033 (6)	0.0028 (6)
C12	0.0135 (8)	0.0196 (8)	0.0232 (8)	0.0037 (6)	−0.0009 (6)	−0.0029 (6)
C13	0.0195 (9)	0.0128 (7)	0.0236 (8)	0.0002 (6)	0.0014 (6)	0.0002 (6)
C14	0.0217 (9)	0.0174 (8)	0.0293 (9)	−0.0002 (7)	−0.0013 (7)	−0.0062 (7)

### Geometric parameters (Å, °)

**Table:**

O1—C3	1.4271 (19)	C5—H5A	0.9600
O1—C2	1.4273 (19)	C5—H5B	0.9600
O2—C7	1.4196 (19)	C5—H5C	0.9600
O2—H2	0.8200	C6—C7	1.526 (2)
O3—C10	1.4214 (19)	C6—H6A	0.9700
O3—C9	1.4252 (19)	C6—H6B	0.9700
O4—C14	1.420 (2)	C7—H7A	0.9700
O4—H4	0.8200	C7—H7B	0.9700
N1—C5	1.501 (2)	C8—C9	1.511 (2)
N1—C6	1.5128 (18)	C8—H8A	0.9700
N1—C1	1.5133 (18)	C8—H8B	0.9700
N1—C4	1.5157 (19)	C9—H9A	0.9700
N2—C12	1.4985 (19)	C9—H9B	0.9700
N2—C11	1.5155 (19)	C10—C11	1.520 (2)
N2—C8	1.5176 (19)	C10—H10A	0.9700
N2—C13	1.5229 (19)	C10—H10B	0.9700
C1—C2	1.515 (2)	C11—H11A	0.9700
C1—H1A	0.9700	C11—H11B	0.9700
C1—H1B	0.9700	C12—H12A	0.9600
C2—H2A	0.9700	C12—H12B	0.9600
C2—H2B	0.9700	C12—H12C	0.9600
C3—C4	1.513 (2)	C13—C14	1.511 (2)
C3—H3A	0.9700	C13—H13A	0.9700
C3—H3B	0.9700	C13—H13B	0.9700
C4—H4A	0.9700	C14—H14A	0.9700
C4—H4B	0.9700	C14—H14B	0.9700
			
C3—O1—C2	109.74 (12)	C7—C6—H6B	108.5
C7—O2—H2	109.5	H6A—C6—H6B	107.5
C10—O3—C9	109.65 (12)	O2—C7—C6	106.26 (12)
C14—O4—H4	109.5	O2—C7—H7A	110.5
C5—N1—C6	109.15 (11)	C6—C7—H7A	110.5
C5—N1—C1	112.03 (11)	O2—C7—H7B	110.5
C6—N1—C1	110.24 (11)	C6—C7—H7B	110.5
C5—N1—C4	111.41 (12)	H7A—C7—H7B	108.7
C6—N1—C4	107.08 (11)	C9—C8—N2	111.81 (12)
C1—N1—C4	106.80 (11)	C9—C8—H8A	109.3
C12—N2—C11	112.05 (11)	N2—C8—H8A	109.3
C12—N2—C8	110.24 (11)	C9—C8—H8B	109.3
C11—N2—C8	107.27 (12)	N2—C8—H8B	109.3
C12—N2—C13	110.16 (12)	H8A—C8—H8B	107.9
C11—N2—C13	110.89 (11)	O3—C9—C8	110.97 (12)
C8—N2—C13	106.02 (11)	O3—C9—H9A	109.4
N1—C1—C2	111.31 (12)	C8—C9—H9A	109.4
N1—C1—H1A	109.4	O3—C9—H9B	109.4
C2—C1—H1A	109.4	C8—C9—H9B	109.4
N1—C1—H1B	109.4	H9A—C9—H9B	108.0
C2—C1—H1B	109.4	O3—C10—C11	111.44 (12)
H1A—C1—H1B	108.0	O3—C10—H10A	109.3
O1—C2—C1	111.36 (13)	C11—C10—H10A	109.3
O1—C2—H2A	109.4	O3—C10—H10B	109.3
C1—C2—H2A	109.4	C11—C10—H10B	109.3
O1—C2—H2B	109.4	H10A—C10—H10B	108.0
C1—C2—H2B	109.4	N2—C11—C10	111.70 (12)
H2A—C2—H2B	108.0	N2—C11—H11A	109.3
O1—C3—C4	111.64 (13)	C10—C11—H11A	109.3
O1—C3—H3A	109.3	N2—C11—H11B	109.3
C4—C3—H3A	109.3	C10—C11—H11B	109.3
O1—C3—H3B	109.3	H11A—C11—H11B	107.9
C4—C3—H3B	109.3	N2—C12—H12A	109.5
H3A—C3—H3B	108.0	N2—C12—H12B	109.5
C3—C4—N1	111.74 (12)	H12A—C12—H12B	109.5
C3—C4—H4A	109.3	N2—C12—H12C	109.5
N1—C4—H4A	109.3	H12A—C12—H12C	109.5
C3—C4—H4B	109.3	H12B—C12—H12C	109.5
N1—C4—H4B	109.3	C14—C13—N2	116.97 (13)
H4A—C4—H4B	107.9	C14—C13—H13A	108.1
N1—C5—H5A	109.5	N2—C13—H13A	108.1
N1—C5—H5B	109.5	C14—C13—H13B	108.1
H5A—C5—H5B	109.5	N2—C13—H13B	108.1
N1—C5—H5C	109.5	H13A—C13—H13B	107.3
H5A—C5—H5C	109.5	O4—C14—C13	113.72 (14)
H5B—C5—H5C	109.5	O4—C14—H14A	108.8
N1—C6—C7	115.07 (12)	C13—C14—H14A	108.8
N1—C6—H6A	108.5	O4—C14—H14B	108.8
C7—C6—H6A	108.5	C13—C14—H14B	108.8
N1—C6—H6B	108.5	H14A—C14—H14B	107.7
			
C5—N1—C1—C2	−68.05 (16)	C12—N2—C8—C9	−69.53 (16)
C6—N1—C1—C2	170.19 (12)	C11—N2—C8—C9	52.72 (16)
C4—N1—C1—C2	54.19 (16)	C13—N2—C8—C9	171.26 (13)
C3—O1—C2—C1	60.35 (16)	C10—O3—C9—C8	61.61 (16)
N1—C1—C2—O1	−59.42 (17)	N2—C8—C9—O3	−58.98 (17)
C2—O1—C3—C4	−59.66 (16)	C9—O3—C10—C11	−61.10 (16)
O1—C3—C4—N1	58.04 (18)	C12—N2—C11—C10	69.22 (16)
C5—N1—C4—C3	69.00 (16)	C8—N2—C11—C10	−51.90 (16)
C6—N1—C4—C3	−171.72 (13)	C13—N2—C11—C10	−167.24 (13)
C1—N1—C4—C3	−53.63 (16)	O3—C10—C11—N2	57.82 (17)
C5—N1—C6—C7	−54.28 (16)	C12—N2—C13—C14	50.60 (17)
C1—N1—C6—C7	69.17 (16)	C11—N2—C13—C14	−74.01 (17)
C4—N1—C6—C7	−175.01 (13)	C8—N2—C13—C14	169.87 (13)
N1—C6—C7—O2	−160.59 (12)	N2—C13—C14—O4	66.19 (18)

### Hydrogen-bond geometry (Å, °)

**Table:**

*D*—H···*A*	*D*—H	H···*A*	*D*···*A*	*D*—H···*A*
O2—H2···Cl2^i^	0.82	2.20	3.0222 (13)	178.
O4—H4···Cl1	0.82	2.24	3.0505 (13)	168.
C1—H1B···Cl1^ii^	0.97	2.64	3.5812 (16)	165.
C2—H2B···O2^iii^	0.97	2.59	3.335 (2)	134.
C4—H4B···Cl2^iv^	0.97	2.72	3.6390 (17)	158.
C5—H5C···Cl1^v^	0.96	2.71	3.6527 (16)	167.
C6—H6A···Cl1^iv^	0.97	2.77	3.6888 (18)	158.
C8—H8A···O2	0.97	2.45	3.385 (2)	163.
C9—H9B···Cl1^iv^	0.97	2.80	3.7625 (17)	174.
C11—H11A···O4	0.97	2.52	3.064 (2)	115.
C13—H13A···Cl2^vi^	0.97	2.80	3.7009 (18)	154.
C13—H13B···O3^vii^	0.97	2.60	3.3013 (19)	130.

Symmetry codes: (i) −*x*+1, *y*−1/2, −*z*+3/2; (ii) −*x*+3/2, −*y*, *z*+1/2; (iii) −*x*+2, −*y*, −*z*+2; (iv) *x*+1/2, *y*, −*z*+3/2; (v) *x*, −*y*+1/2, *z*+1/2; (vi) −*x*+3/2, *y*−1/2, *z*; (vii) −*x*+2, *y*−1/2, −*z*+3/2.
